# Investigating the association of environmental exposures and all-cause mortality in the UK Biobank using sparse principal component analysis

**DOI:** 10.1038/s41598-022-13362-3

**Published:** 2022-06-02

**Authors:** Mohammad Mamouei, Yajie Zhu, Milad Nazarzadeh, Abdelaali Hassaine, Gholamreza Salimi-Khorshidi, Yutong Cai, Kazem Rahimi

**Affiliations:** Deep Medicine, Nuffield Department of Women’s & Reproductive Health, Oxford Martin School, University of Oxford, 1st Floor, Haye House, 75 George Street, Oxford, OX1 2BQ UK

**Keywords:** Risk factors, Environmental impact, Statistical methods

## Abstract

Multicollinearity refers to the presence of collinearity between multiple variables and renders the results of statistical inference erroneous (Type II error). This is particularly important in environmental health research where multicollinearity can hinder inference. To address this, correlated variables are often excluded from the analysis, limiting the discovery of new associations. An alternative approach to address this problem is the use of principal component analysis. This method, combines and projects a group of correlated variables onto a new orthogonal space. While this resolves the multicollinearity problem, it poses another challenge in relation to interpretability of results. Standard hypothesis testing methods can be used to evaluate the association of projected predictors, called principal components, with the outcomes of interest, however, there is no established way to trace the significance of principal components back to individual variables. To address this problem, we investigated the use of sparse principal component analysis which enforces a parsimonious projection. We hypothesise that this parsimony could facilitate the interpretability of findings. To this end, we investigated the association of 20 environmental predictors with all-cause mortality adjusting for demographic, socioeconomic, physiological, and behavioural factors. The study was conducted in a cohort of 379,690 individuals in the UK. During an average follow-up of 8.05 years (3,055,166 total person-years), 14,996 deaths were observed. We used Cox regression models to estimate the hazard ratio (HR) and 95% confidence intervals (CI). The Cox models were fitted to the standardised environmental predictors (a) without any transformation (b) transformed with PCA, and (c) transformed with SPCA. The comparison of findings underlined the potential of SPCA for conducting inference in scenarios where multicollinearity can increase the risk of Type II error. Our analysis unravelled a significant association between average noise pollution and increased risk of all-cause mortality. Specifically, those in the upper deciles of noise exposure have between 5 and 10% increased risk of all-cause mortality compared to the lowest decile.

## Introduction

Numerous studies have reported significant associations between individual environmental variables such as traffic noise, air pollution, green space and health outcomes^1–4^. Such findings are important but a key limitation of them is that they do not consider simultaneous exposure to key environmental stressors in the analysis. Since environmental variables are often highly correlated, this limitation can diminish the causal plausibility of the findings. For instance, multiple studies have reported significant associations between traffic noise, all-cause mortality and cardiovascular diseases^5–7^. However, exposure to higher levels of traffic noise, also increases the likelihood of exposure to particulate matter pollutants (PM), gaseous pollutants and other traffic-related stressors. Additionally, individuals exposed to these stressors are less likely to have access to domestic and urban green spaces which have been reported to have protective effects against adverse health outcomes.

The aforementioned gap in simultaneous analysis of multiple environmental stressors is partly due to “multicollinearity”. Environmental variables such as green space, gaseous and particulate air pollution, noise pollution and traffic-related variables are usually temporally and/or spatially correlated; they are also often correlated with demographic and socioeconomic determinants. The inclusion of these correlated variables in regression models leads to erroneous estimation of the effect size, broad confidence intervals, and therefore, inaccurate interpretation. Methods for mitigating the effects of multiple correlated variables include dimensionality reduction (e.g. Principal Component Analysis (PCA)), partial least-squares, shrinkage regression models, mixture models, and Bayesian approach. However, several factors such as complexity of application, difficulty of interpretation, and high computational requirements have impeded their adoption in environmental research^8,9^. PCA and PLS are not interpretable^10,11^. Shrinkage regression models achieve sparsity by penalising nonzero model coefficients as well as regression error. While this mitigates multicollinearity, penalising regression coefficients has unfortunate implications for statistical inference where the aim is finding reliable estimates of model coefficients regardless of their contribution to predictive performance. Mixture models and Bayesian modelling are computationally demanding and become intractable as the number of variables and observations increase^12,13^. Therefore, a widely applicable, interpretable and computation-efficient statistical approach is needed to fill this gap.

The computational efficiency and desired statistical properties of PCA make it good candidate for big data studies where multicollinearity poses a problem. PCA transforms a group of correlated variables into a smaller group of independent variables, called principal components. Therefore, the use of principal components -instead of the set of variables- in regression analysis eliminates multicollinearity. Due to these advantages, the method has been widely used in epidemiological studies^14–17^. Yet, the main shortcoming of PCA is interpretability. Each principal component is a mix of all variables, making inference impossible. Since this difficulty is the result of a dense transformation, we hypothesise that a sparse transformation could facilitate the interpretation of findings. To this end, we investigated the usefulness of Sparse Principal Component Analysis (SPCA)^18^. As a case study, we focused on potentially modifiable but correlated environmental exposures. As such, the main contributions of the study are, firstly, we showcase the benefits of SPCA as an interpretable alternative to PCA, offering clear advantages for statistical inference in the presence of multicollinearity. Secondly, using SPCA, we showed a significant association between noise levels and all-cause mortality after adjusting for a comprehensive list of correlated environmental variables that could affect health outcomes independent of noise levels, namely residential traffic levels, vicinity to roads and major roads, green space, natural environment, domestic garden, proximity to water, and coastal proximity. While several studies in the past decade have largely addressed the questions around the confounding effects of air pollution and traffic noise^5–7,19^, none of the available studies have adjusted for the comprehensive list of environmental confounders considered in our study.

## Methods

### Study population

This analysis was conducted using the UK Biobank cohort. The UK Biobank is a large prospective cohort study involving 502,527 participants aged 40–69 years who were recruited between 2006 and 2010 from 22 assessment centres across the UK^20^. The data is globally accessible to approved researchers. We excluded participants from analysis if they had any of the following: (a) withdrawal of consent for future data linkage from the UK Biobank after recruitment (158 individuals) (b) left the UK (1102 individuals) (d) deaths reported by relatives but not recorded in death registry data (38 individuals) (e) missing data on investigated environmental exposures (69,268 individuals) and (f) change of residential address after the baseline (52,271 individuals). The final sample size consisted of 379,690 individuals. All participants provided written consent, ethical approval was obtained from the North West Multi-Centre Research Ethical Committee and Patient Information Advisory Group and all methods were performed in accordance with the relevant guidelines and regulations.

### Environmental exposures

Measures for exposure to air pollutants included: the annual average concentration of PM_2.5_, PM_10_, and PM_coarse_ (particulate matter (PM) with an aerodynamic diameter of less than 2.5 µm, 10 µm, and between 2.5 and 10 µm respectively), NO_2_ (nitrogen dioxide), and NO_x_ (nitrogen oxides). These measures were calculated for year 2010 for each participant’s residential address at recruitment using Land Use Regression model developed and validated by the ESCAPE project^21,22^.

Exposures to traffic were also derived by the ESCAPE project for each participant's home: traffic intensity on the nearest road, traffic intensity on the nearest major road (*traffic intensity*, vehicles/day), and sum of major road length within 100 m buffer. Average daytime, evening time and night-time sound level of road traffic noise pollution were derived for year 2010 using the CNOSSOS model^23^.

Other environmental indicators included the proportion of green space, natural environment, domestic garden, and water within 300 m and 1000 m of residential addresses, using the 2005 Generalised Land Use Database for England and Centre for Ecology and Hydrology 2007 Land Cover Map data for Great Britain^24^. The buffer sizes were decided based on relevant health evidence and public policy on both density and accessibility. Coastal proximity was estimated using Euclidean distance raster^25^.

All the exposure indicators were only modelled or available to a single year, which may differ up to 4 years from recruitment. This may particularly affect air pollution and road traffic noise estimates, distributions of which tend to be spatially and temporally different. As with other studies^26,27^ using these air pollution and noise data in UK Biobank, we made an assumption that whilst the absolute traffic volumes will have changed between earlier baseline periods and 2010, the relative difference in these exposures would likely have been spatially stable over this short period in the UK. This assumption is supported by findings for NO_2_ air pollution in Great Britain, for which road traffic is a major source, where LUR-modelled NO_2_ estimates for 2009 could be reliably back-extrapolated to earlier 1990s^28^. Between years 2010 and 2018, total annual emissions for PM_10_ and PM_2.5_ have been stable across the UK while emissions for NO_2_ have proportionally decrease according to the official statistics^29^. While we cannot exclude the possibility of exposure misclassification, the decision of using single-year annual average exposures at baseline to represent the annual average exposures during the entire follow-up period was deemed justifiable.

### Additional covariates

In the regression analysis, we adjusted for a number of sociodemographic, socioeconomic, physiological, behavioural and lifestyle determinants of health. Specifically, we adjusted for age, sex, ethnicity, Townsend Deprivation Index, household income, qualifications, employment status, standing height, body mass index, average systolic blood pressure (SBP), average diastolic blood pressure (DBP), average pulse rate (PR), alcohol consumption and smoking status. Table 1 provides a descriptive summary of the cohort.

**Table 1:** Descriptive summary of the study sample, environmental exposures, and outcome.

Variables	Women (*n* = 206,925)	Men (*n* = 172,765)	All (*n* = 379,690)
Age, mean (SD)	56.66 (7.94)	57.15 (8.10)	56.88 (8.02)
Age at the time of event (SD)	66.39 (7.32)	67.28 (6.87)	66.93 (7.06)
Townsend deprivation index, mean (SD)	− 1.44 (2.96)	− 1.39 (3.06)	− 1.42 (3.01)
Ethnicity: British (%)	87.98	88.84	88.37
Ethnicity: Any other white background (%)	3.56	2.60	3.12
Ethnicity: Irish (%)	2.46	2.67	2.55
Ethnicity: Indian (%)	1.20	1.37	1.28
Ethnicity: other (%)	3.53	4.43	4.58
Annual average day-time noise level (dB(A))a, mean (SD)	55.35 (4.22)	55.39 (4.27)	55.37 (4.24)
Annual average evening noise level (dB(A))^a^, mean (SD)	51.61 (4.22)	51.64 (4.27)	51.62 (4.24)
Annul average night-time noise level (dB(A))^a^, mean (SD)	46.53 (4.22)	46.57 (4.27)	46.55 (4.24)
Domestic garden coverage (%) within 1000m^b^, mean (SD)	24.46 (11.26)	24.24 (11.23)	24.36 (11.25)
Domestic garden coverage (%) within 300m^b^, mean (SD)	31.49 (14.67)	31.27 (14.70)	31.39 (14.68)
Greenspace coverage (%) within 1000m^c^, mean (SD)	45.28 (21.56)	45.37 (21.44)	45.32 (21.51)
Greenspace coverage (%) within 300m^c^, mean (SD)	35.40 (23.20)	35.51 (23.07)	35.45 (23.14)
Natural environment coverage (%) within 1000m^d^, mean (SD)	41.32 (25.67)	41.35 (25.59)	41.33 (25.63)
Natural environment coverage (%) within 300m^d^, mean (SD)	26.68 (25.31)	26.78 (25.25)	26.72 (25.28)
Water body coverage (%) within 1000m^e^, mean (SD)	1.24 (2.46)	1.25 (2.45)	1.25 (2.46)
Water body coverage (%) within 300m^e^, mean (SD)	0.87 (2.88)	0.89 (2.90)	0.88 (2.89)
Costal distance (meter), mean (SD)	45.39 (26.82)	45.85 (26.77)	45.60 (26.80)
NO_2_; (μg/m^3^), mean (SD)	26.67 (7.50)	26.72 (7.58)	26.69 (7.54)
NO_x_; (μg/m^3^), mean (SD)	43.89 (15.21)	44.05 (15.55)	43.96 (15.36)
PM_10_; (μg/m^3^), mean (SD)	16.22 (1.87)	16.23 (1.88)	16.23 (1.87)
PM_coarse_; (μg/m^3^)^f^, mean (SD)	6.42 (0.89)	6.42 (0.89)	6.42 (0.89)
PM_2.5_; (μg/m^3^), mean (SD)	9.98 (1.03)	9.99 (1.05)	9.98 (1.04)
Sum of major road length within 100 m (m) ^g^, mean (SD)	27.25 (75.41)	28.23 (77.80)	27.70 (76.51)
Traffic intensity on nearest major road (vehicles/day)^h^, mean (SD)	23,472.94 (21,322.17)	23,477.37 (21,272.41)	23,474.95 (21,299.52)
Traffic intensity on nearest road (vehicles/day)^h^, mean (SD)	1480.04 (4906.16)	1516.51 (5020.38)	1496.63 (4958.49)
Years of follow-up, mean (SD)	8.08 (1.03)	8.01 (1.19)	8.05 (1.10)
Number of events	5954 (2.88%)	9042 (5.23%)	14,996 (3.95%)
Incidence rate, per 1000 person-years	4	7	5

^a^Average sound level pressure LAeq between the hours of 07:00 to 19:00 for day-time; 19:00–23:00 for evening; 23:00–07:00 for night-time;

^b^Derived from the land use types classed as 'domestic garden' from the Generalised Land Use Database (GLUD) 2005 for England at the Census Output Area level;

^c^Derived from the land use types classed as 'greenspace' from the Generalised Land Use Database (GLUD) 2005 for England at the Census Output Area level;

^d^Derived from the land cover classified as 'natural environment' from the Land Cover Map (LCM) 2007;

^e^Derived from the land use types classed as 'water' from the Generalised Land Use Database (GLUD) 2005 for England at the Census Output Area level;

^f^PM coarse (particulate matter between 2.5 and 10 µm); Land Use Regression (LUR) estimate for annual average 2010;

^g^The definition of a major road for the local road network is a road with traffic intensity greater than 5000 motor vehicles per 24 h;

^h^Traffic intensity is the average total number of motor vehicles per 24 h on the nearest major road based upon a local road network.

### Health outcome

**Figure 1:**
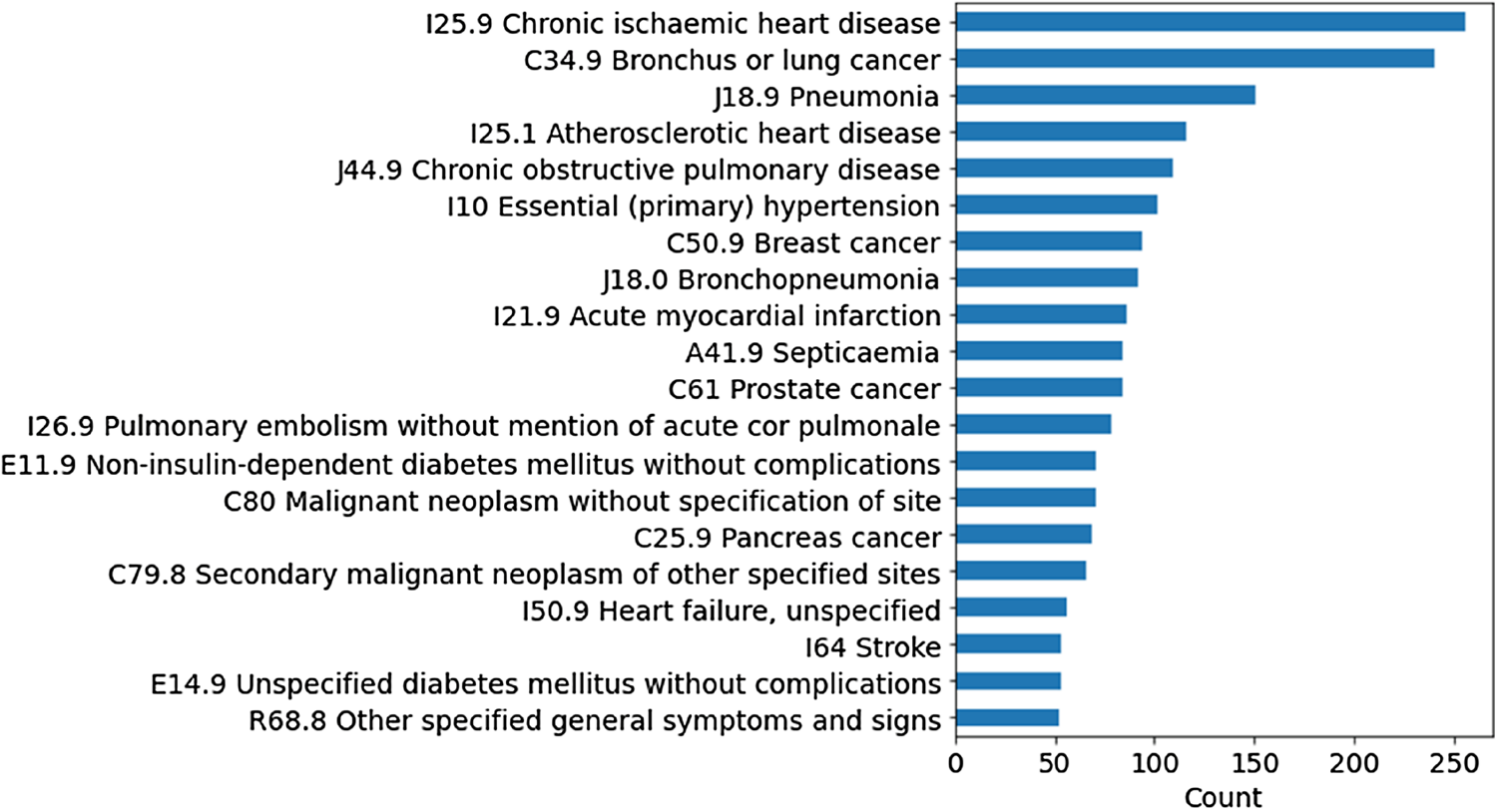
The top 20 primary causes of deaths within the cohort.

We used all-cause mortality as the outcome of interest. The date of death was extracted from the linked national death registries. An event was ascertained if death was recorded between the date of recruitment and the end of follow-up (censoring date: 1st May 2017). Fig. 1. shows the top 20 ICD10 codes that were registered as the primary causes of death.

### Statistical analysis

**Figure 2:**
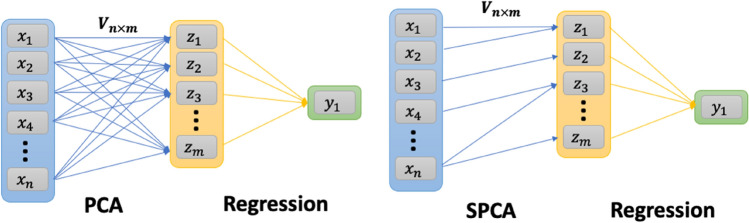
Schematic representation of PCA and Sparse PCA projection of the variables (**x**) to the latent space or principal components (**z**). The second layer shows a subsequent regression analysis for the outcome of interest (**y**).

We used SPCA, which was originally proposed by Zou and colleagues^18^. Our hypothesis is that the sparsity of principal components in SPCA can help overcome the limitation of PCA for identifying important stressors. The term ‘sparse’ in SPCA means that most of the coefficients in the loading matrix will be zeros, thus each derived principal components in SPCA will only be related to a small subset of the variables. Additionally, in contrast to PCA, each variable can only contribute to a small numbers of principal components in SPCA. These two features are expected to facilitate the interpretability of results. This is schematically demonstrated in Fig. 2, where **x**_**i**_ $\in$ ${\mathbb{R}}^{{\varvec{n}}}$ is the vector of variables for the observation $i$. The arrows represent the loading matrix **V** $\in$ ${\mathbb{R}}^{{{\varvec{n}} \times {\varvec{m}}}}$ and map the variables to principal components **z**_**i**_ $\in$ ${\mathbb{R}}^{{\varvec{m}}}$ where often m $\ll n$_**.**_ Following this projection, a regression analysis may map the principal components to the outcome of interest, **y.** Standard statistical hypothesis testing methods can determine the significance of associations between the principal components, **z,** and the outcome, **y,** however, the dense mapping between the variables, **x,** and the principal components, **z**, mean these associations cannot be traced back to the variables. We expect SPCA to resolve this by providing a sparse loading matrix.

In order to achieve sparsity, SPCA penalizes the absolute value of the loadings at the cost of loss of information. A hyper-parameter, $\lambda$, is used to balance the trade-off between information loss and the sparsity of the loading matrix. Several implementations of SPCA have been proposed, here, we used the implementation reported by Erichson et al.^30^ which uses the following formulation:

$$\mathop {\min }\limits_{B} v\left( B \right) + { }\psi \left( B \right),$$

where,

$$v\left( B \right): = \mathop {\min }\limits_{A} \frac{1}{2} \left\| {X - XBA^{T} } \right\|_{F}^{2} {\text{subject to}}A^{T} A = I ,$$

$v\left( B \right)$ represents the reconstruction error, $\psi \left( B \right)$ is the penalty term which could be L1 norm (LASSO), L2 norm (RIDGE), or a combination of the two (elastic net). The hyperparameter λ controls the trade-off between the reconstruction error and sparsity; a larger value of λ produces a sparser model. Hereafter this parameter is denoted by $\lambda_{SPCA}$ to distinguish it from the penalty coefficient in the penalised regression model ($\lambda_{Cox}$). The data matrix is denoted by X, B is the sparsely weighted matrix and A is an orthonormal matrix.

Data processing, modelling and visualisations were performed in Python v.3.8.3 and R v.4.1.0. Cox models and related plots were obtained using the Python library Lifelines v.0.25.10, the PCA was performed using the package scikit-learn v.0.25.10 and SPCA using the SPCA R library^30^. Sankey plots were obtained using Plotly v.5.3.1.

## Results

We used Cox regression to evaluate the association of environmental variables with all-cause mortality after adjusting for the aforementioned covariates. We compared the results when,

- the environmental variables were plugged into the model with no transformation (***Cox model*** hereafter).
- L1 penalty was included in the model (***penalised*** ***Cox*** ***model*** hereafter). We varied the coefficient of the L1 penalty term, $\lambda_{Cox}$, between 0 and 2e-3 at 5e-5 intervals producing different levels of sparsity (supplementary materials: Fig. S1).
- The environmental variables were transformed with PCA. The number of principal components was selected to explain 90% of the variance in the data, leading to seven principal components. The Cox regression model was fitted to the resulting principal components and other covariates (***PCA Cox model*** hereafter);
- We repeated step (c) using SPCA. The coefficient of the L1 penalty, $\lambda_{SPCA}$, was selected to increase model parsimony. Increasing the value of $\lambda_{SPCA}$ results in principal components that consist of a smaller set of variables. To facilitate interpretability, we selected $\lambda_{SPCA}$ such that no two principal components share the same variable, in other words each variable at most contributes to one principal component. More details about the selection of $\lambda_{SPCA}$ is included in supplementary materials (Fig. S2). The number of principal components were similarly selected to explain 90% of the data variance, leading to seven principal components (S***PCA Cox model*** hereafter).

The number of follow-up years was the underlying time variable for all Cox models. Prior to the analysis, all numeric variables were examined for normality and outliers. Subsequently, they were standardised and values above or below five, were set to five.

**Figure 3:**
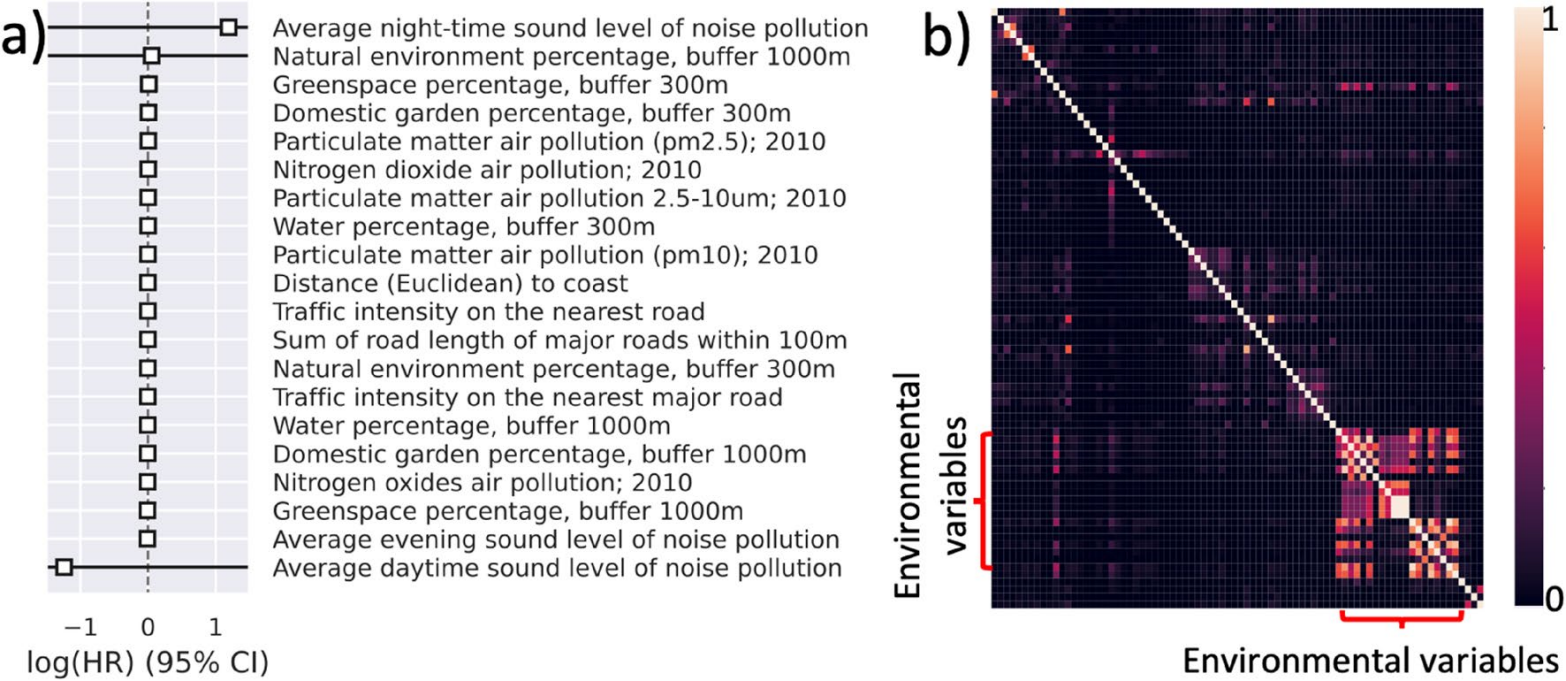
(**a**) The plot shows log(HR) per 1 standard deviation increase of the variables (**b**) Pairwise Pearson correlation between socioeconomic, demographic, physiological and environmental factors in a large cohort of 379,690 in the UK.

Figure 3a depicts the coefficient of the environmental variables in the Cox model. Multicollinearity in the Cox model results in high standard errors in the estimation of the coefficients, inhibiting reliable statistical inference. None of the environmental variables are found to be statistically significant. The detailed results are included in supplementary materials (Table S1). Figure 3b shows pairwise Pearson correlation between the variables. The block with high correlation coefficients pertains to the 20 environmental variables, underlining high collinearity within this class of variables. A moderate correlation is also observed between Townsend deprivation index and a number of environmental variables. A larger figure with detailed labels is included in supplementary materials (Fig. S3).

**Figure 4:**
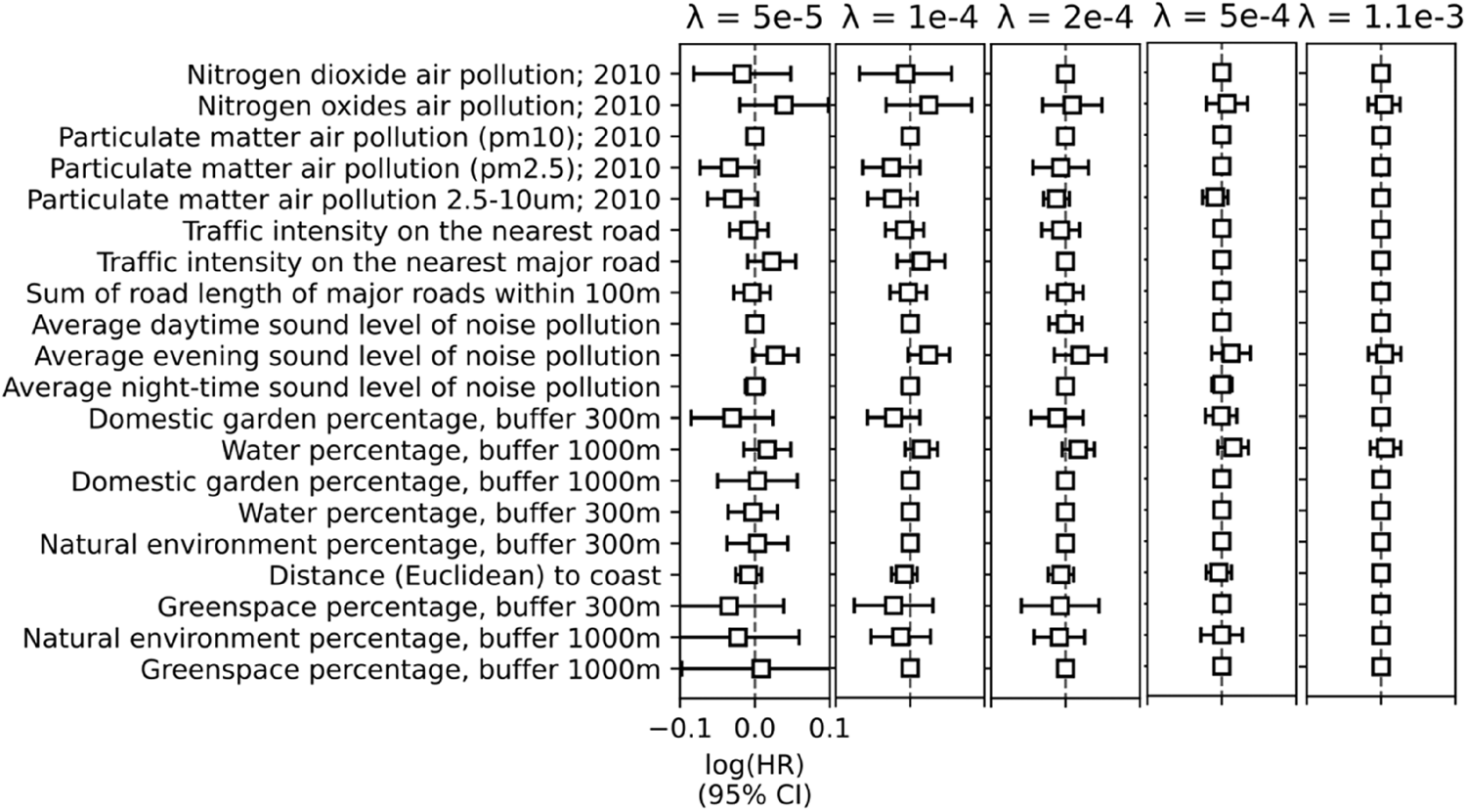
Log(HR) for different values of the L1 penalty coefficient (${\lambda }_{Cox}$) in the penalised Cox model.

Adding the L1 penalty (penalised Cox model) attenuates the log(HR) associated with the environmental variables. Large values of $\lambda_{Cox}$ result in log(HR) = 0 for all environmental variables. None of the intermediate values of $\lambda_{Cox}$ produced any log(HR) values significantly different from zero at α = 5%. Lastly, similar to the Cox model without L1 penalty, multicollinearity led to convergence errors for several smaller values of $\lambda_{Cox}$. Figure 4 demonstrated the shrinkage of the log(HR) estimates and the 95% CI for different values of $\lambda_{Cox}$.

**Figure 5:**
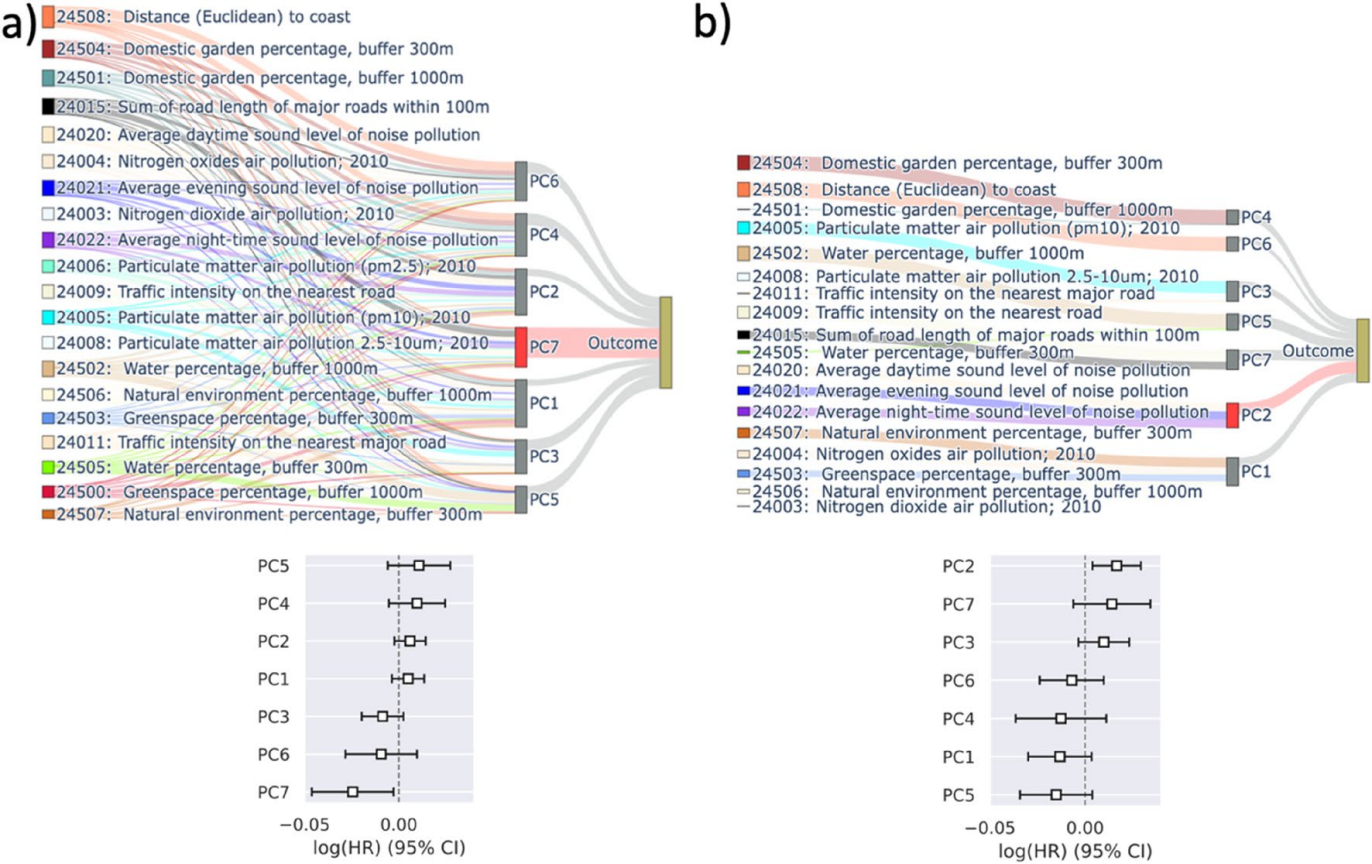
Schematic representation of the association of environmental variables with all-cause mortality using a two-stage regression analysis (**a**) with PCA and (**b**) with SPCA. In the first stage (i.e. dimensionality reduction), the variables are transformed to principal components. In the second stage, a Cox model was used to investigate the association of the transformed variables and all-cause mortality.

Figure 5, schematically compares PCA and SPCA results. The environmental variables are shown in the far left. The width of the links between the variables and the principal components are proportional with the loading coefficients. The links between the principal components and the outcome (i.e. all-cause mortality) are similarly proportional with the absolute value of the Cox coefficients (log(HR)). The associations that were found significant at α = 5% are highlighted in red. Detailed results are included in supplementary materials (Table S2).

In the PCA Cox model, the seventh component has a negative association with the outcome, however, given the complex interrelationship between the variables and principal components, it is not possible to disentangle this association. On the contrary, in the SPCA Cox model, the second component has a positive association with mortality and this can be easily traced back to the three constituting variables of this component. Specifically, this component is the average of the three variables representing average level of sound pollution in daytime, evening, and night-time. One unit change in this principal component, corresponds to 2.47 dB increase in the average daily noise pollution (details in supplementary materials) and this is associated with HR:1.017 (95% CI: 1.004–1.030). Although the list of covariates that we adjusted for is much more comprehensive than previous studies and included some traffic-related stressors that were correlated with noise pollution, our result (HR: 1.07, 95%CI: 1.02–1.13, per 10 dB increase in the average daily noise) is in agreement with the previous studies^5,7^.

**Figure 6:**
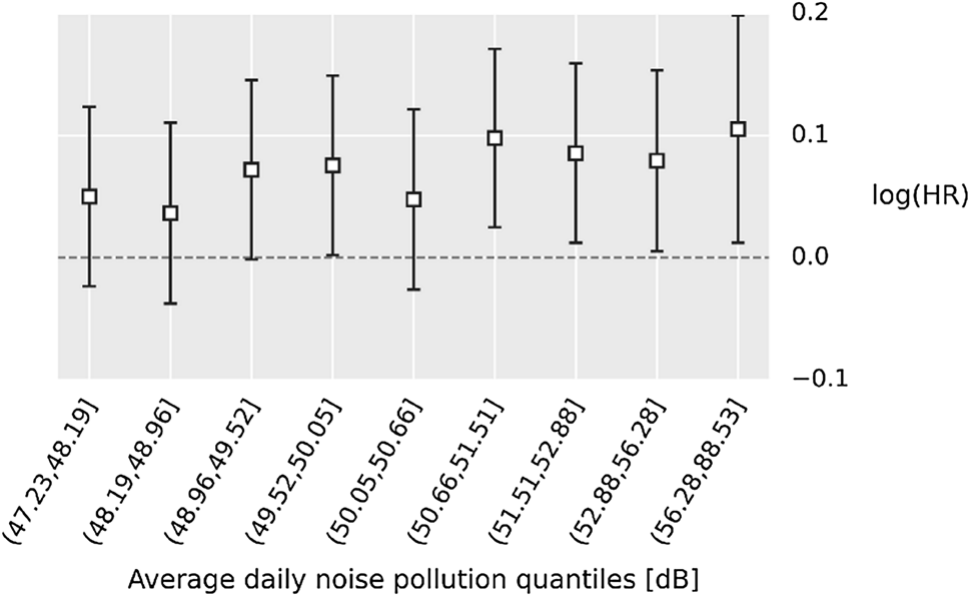
Log(Hazard Ratio) of all-cause mortality for different noise pollution exposure deciles compared to the lowest decile, i.e. (46.72, 47.23], after adjusting for socioeconomic, demographic, environmental, and physiological, and behavioural covariates.

To further verify this association, we investigated whether it persists across different exposure levels. To this end, as suggested by the previous analysis, the three aforementioned noise pollution variables were averaged; forming a new variable that represents average daily noise pollution. This was then categorised into deciles and the hazard ratios were calculated for the nine top deciles relative to lowest decile. The lowest decile represents noise pollution levels between 46.72 and 47.23 dB. To address the multicollinearity of the environmental covariates, the remaining 17 environmental variables were transformed to principal components explaining 0.92 percent of the variance. The model was adjusted for all other covariates. The results are depicted in Fig. 6, showing an upward trend which underlines the plausibility of a causal link. Descriptive summary of the subpopulations in each category and more details about the model is included in the supplementary materials (Table S3 and 4).

## Discussion

### Main findings

The key finding of this study is the integration of SPCA in regression analysis provides a promising approach for conducting inference in the presence of multicollinearity. Multicollinearity leads to erroneous estimation of the coefficients and broad confidence intervals in regression models. This increases the likelihood of Type II errors. As a result, some important associations may remain concealed. As a case study, we investigated the association of various correlated environmental factors and all-cause mortality, adjusting for other established risk factors. SPCA resulted in a sparse and interpretable grouping of the environmental variables. For instance, all three noise related predictors were combined into one principal component. Additionally, the sparsity of the transformation enabled us to trace the statistical significance of the principal components back to the variables of interest.

### Interpretation of findings in the context of previous studies

PCA, as a descriptive analysis tool is one of the oldest and most commonly used techniques for reducing the dimensionality of data^31^. But the lack of interpretability of the derived representations, i.e. principal components, has been recognised as one of its major drawbacks. As shown in our results, in PCA, the entangled relationship between the principal components and the variables hinders the interpretation of findings. While some seek to mitigate this issue by deselecting non-important variables^32^ or selecting variables more relevant to outcomes using supervised methods^33,34^, such interventions are not appropriate for statistical hypothesis testing where all relevant covariates should be adjusted for regardless of their contribution to predictive performance.

Over the years, other dimensionality reduction methods have been widely applied in different disciplines. Random Projection^35^, Dictionary Learning^36^, Factor Analysis, Independent Component Analysis^37^, Non-negative Matrix Factorization (NMF)^38^ are examples of these methods. Recently, Autoencoders including Denoising Autoencoder^39^ and Variational Autoencoder^40^ are increasingly used to learn a low dimensional representation of the input variables. But similar to PCA, the common limitation of these dimensionality reduction methods is the entangled relationship between the variables and the low dimensional representations. Enforcing sparsity in the transformation is recognised as an effective way to address this problem^41^. Inspired by this we investigated the use of SPCA for statistical hypothesis testing in the context of environmental health research and showed promising results. In light of the findings, we conclude that the integration of SPCA in statistical inference is a simple, computationally-efficient strategy for big data investigations when multicollinearity could lead to erroneous results.

Previously, environmental epidemiology studies have adopted dimensionality reduction methods, as well as one-stop methods such as Bayesian profile regression^42^ to perform both dimensionality reduction and regression analysis for multiple pollutants. Some studies using PCA had previously identified a subset of air pollutants that were associated with mortality^34,43^. However, no studies have applied these statistical techniques to adjust for the wide range of environmental and non-environmental covariates that we considered in our analysis^44^. Neighbourhood-wide^45^ and environment-wide^24^ association studies (N/EWAS) have also been applied to high dimensional data in environmental epidemiology. These methods are inspired by genome-wide association studies^46^ and their resources-intensiveness -in terms of data and computational power- hinders their wider adoption.

Our analysis led to a clear pattern of association between noise pollution and all-cause mortality. Noteworthy, the three indicators of noise pollution, day-time, evening, and night time noise levels, were combined into one principal component, all with the same weights. The resulting principal component (or a 2.47 dB increase in the average daily noise pollution) was associated with a HR:1.017 (95%CI:1.004–1.030) for all-cause mortality. This is translated to a HR: 1.07, 95%CI: 1.02–1.13 as per 10 dB increase in average noise level, in line with the only other study that showed a positive significant association between daily road traffic noise exposure and all-cause mortality (HR: 1.08, 95%CI: 1.04–1.12)^5^. A previous study in London reported the association between daytime noise and all-cause mortality in areas with noise pollution level greater than 60 dB compared to areas with noise pollution level less than 55 dB RR: 1.04 (95%CI: 1.00–1.07)^7^. While the hazard ratio calculated in our study is not directly comparable to the aforementioned studies, due to differences in the populations, study designs, data processing and covariates, the consistency of the findings are reassuring. Although the inclusion of correlated covariates can attenuate the significance of association, our results are largely in agreement with these studies, suggesting independent of gaseous pollutant, traffic-related stressors and other determinants, noise level is an important risk factor. Nonetheless, number of studies investigating the epidemiological link between road traffic noise exposure and all-cause mortality outcomes remains few, with a recent meta-analysis showing a weak association by pooling only five studies (HR: 1.01, 95%CI: 0.98–1.05)^47^.

A subsequent exposure–response analysis showed that the four highest exposure deciles are associated with significant risk of all-cause mortality compared to the lowest exposure decile. Specifically, the top four average daily noise exposure deciles (50.66, 51.51], (51.51, 52.88], (52.88, 56.28], (56.28,88.53] dB were consistently associated with significant increase in all-cause mortality of HR:1.10 (95%CI: 1.03–1.19), 1.09 (95%CI: 1.01–1.17), 1.08 (95%CI: 1.01–1.17), 1.11 (95%CI: 1.01–1.22) compared to the lowest decile of (46.23, 47.23] dB. It should be noted that, 8 of the top 20 causes of death included in this study were cardiometabolic diseases. The finding of our trend analysis is similar to what was previously reported for the cardiovascular disease mortality, suggesting a possible effect threshold may start at around 50–53 dB^47–49^.

### Limitations and future works

The key strengths of our analysis are, a large cohort, adjustment for a comprehensive list of environmental exposures, including correlated traffic-related exposures, which was facilitated by our methodological approach. This study has limitations. Firstly, we did not account for any potential non-linear exposure–response relationship. The inclusion of non-linear and interaction terms could reduce the risk of residual confounding. However, our primary objective was to study the usability of SPCA as a simple, computationally efficient and interpretable method to address collinearity. Second, as we already noted, exposure misclassification is inevitable for this type of study. Typically, if there was a true association with the health outcome, the effect estimates would be biased toward null for a classic random error. Third, SPCA approach is essentially a data-driven method without a priori variables hypotheses, without considering causal structures among the variables and/or variable-outcome links. In our study, 20 environmental exposures from UK Biobank were reduced in dimensionality using SPCA and were all used in the Cox regression under the assumptions of a causal structure linking each exposure and the outcome and the assumption that the exposures are independent of one another. However, in reality, some exposures may be on a specific causal pathway (e.g. traffic intensity–air pollution–mortality). It is beyond the scope of current study to investigate this complex causal structure which indeed requires a careful consideration of the causal inference analysis framework. Taking together all these limitations, the findings generated from our SPCA analysis are mainly exploratory and neither infers any potential causal relationship nor biological plausibility.

## Conclusion

This study demonstrated that SPCA is a viable analytical approach to address, and enable interpretability of multiple environmental stressors-health associations. Using this method, our study further verified existing evidence on the association between noise as an important risk factor for adverse health outcomes in the UK Biobank. The strength of our analysis was observing this association even after adjusting for comprehensive list correlated stressors.

## Supplementary Information

Supplementary Information.

## Data Availability

The data that support the findings of this study are available from the UK Biobank but restrictions apply to the availability of these data, which were used under license for the current study. The raw data are only available to approved researchers via the UK Biobank.

## Abbreviations

CI: Confidence Interval
CNOSSOS-EU: Common Noise Assessment Methods in Europe
CVD: Cardiovascular disease
ESCAPE: European Study for Cohorts of Air Pollution Effects
HR: Hazard ratio
ICD: International classification of diseases
LUR: Land Use Regression
N/EWAS: Neighbourhood-wide or environment-wide associations
NMF: Non-negative matrix factorisation
PC: Principal component
PM: Particulate matter
PCA: Principal component analysis
SD: Standard deviation
SPCA: Sparse principal component analysis
