# Supplementary material for: Investigating the association of environmental exposures and all-cause mortality in the UK Biobank using sparse principal component analysis
Source: Sci Rep. 2022 Jun 2;12:9239. doi: 10.1038/s41598-022-13362-3 (PMC9163152; doi:10.1038/s41598-022-13362-3)
Supplement: Supplementary file 1 — Supplementary Information. [file 41598_2022_13362_MOESM1_ESM.docx]

**Investigating the association of environmental exposures and all-cause mortality in the UK Biobank using sparse principal component analysis**

Mohammad Mamouei, Yajie Zhu, Milad Nazarzadeh, Abdelaali Hassaine, Gholamreza Salimi-Khorshidi, Yutong Cai and Kazem Rahimi

**Corresponding author**: Dr Mohammad Mamouei, Deep Medicine, Nuffield Department of Women’s & Reproductive Health, Oxford Martin School, 1^st^ Floor, Haye House, 75 George Street, Oxford, OX1 2BQ, United Kingdom. Email: Mohammad.Mamouei@wrh.ox.ac.uk

All authors are affiliated with Deep Medicine, Nuffield Department of Women’s & Reproductive Health, Oxford Martin School, University of Oxford

**Selection of λ:**

The parameter λ was varied across a range of values and the number of principal components were adjusted such that at least 90% of the variance is explained. Figure below depicts the results. The maximum number of nonzero loadings represents the maximum number of variables that constitute one of the principal components. The maximum number of variables in principal component (Max #variables in PCs), represents the maximum number of principal components that a variable has contributed to. The larger values of λ lead to more sparse representations. We selected λ such that each variable at most contributes to one principal component.

**Figure S1. Shrinkage of the log(HR) for environmental variables as a function of the coefficient of the L1 penalty (**$\boldsymbol{\lambda}$**). The value of** $\boldsymbol{\lambda}$ **was varied between 0 to 2e-3 with 5e-05 steps. Similar to Cox model, in the penalised Cox models, several values of** $\boldsymbol{\lambda}$ **produced convergence errors due to multicollinearity these values are displayed below.**


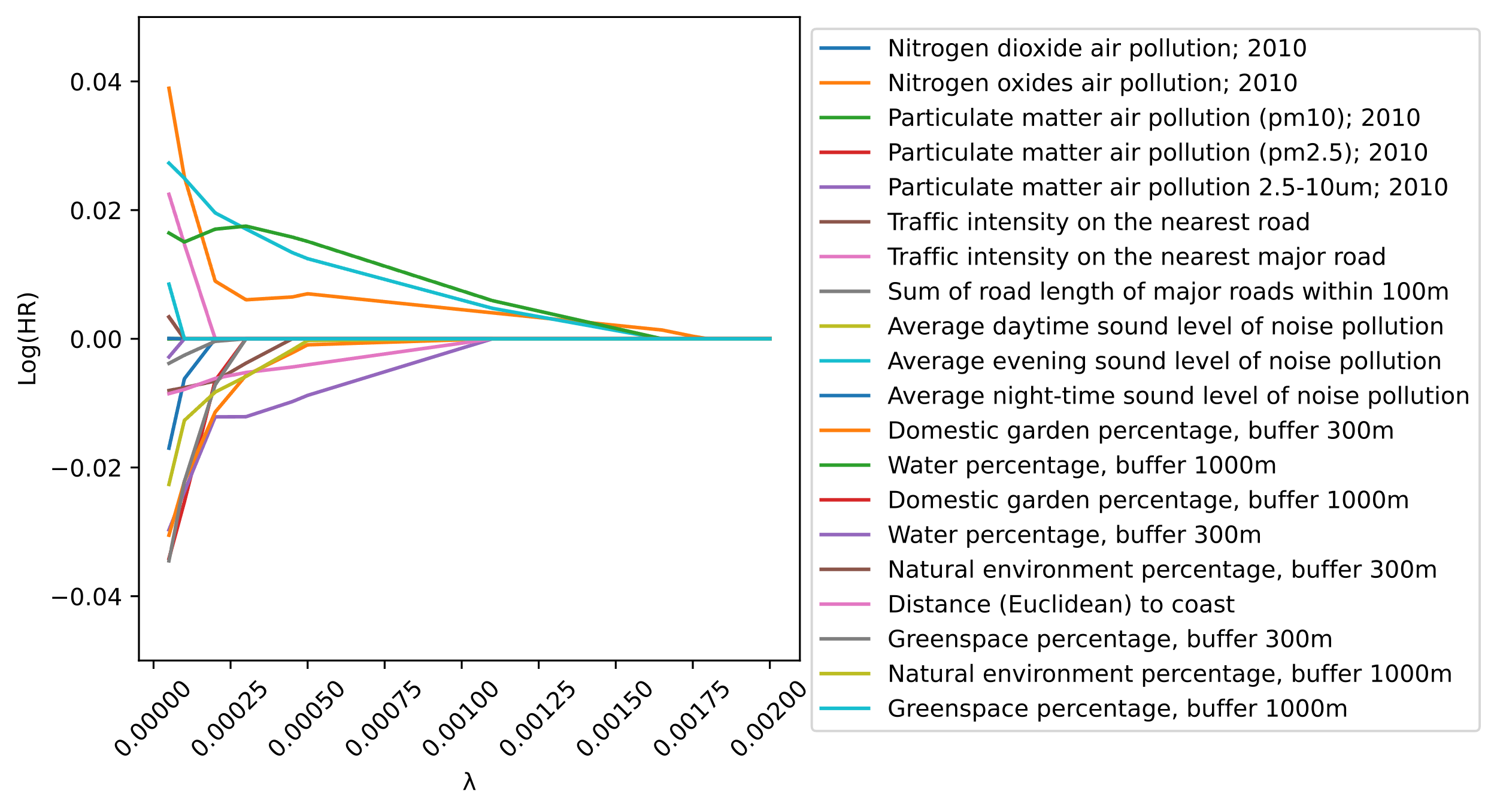


**Figure S2. Changes in the maximum number of nonzero loadings (Max #nonzero loadings), the maximum number of PCs that any variable has contributed to (Max #variables in PCs), and the number of PCs as a function of the regularisation parameter Lambda. In all instances the smallest number of PCs was selected in order to explain at least 90% of the data variance.**

**
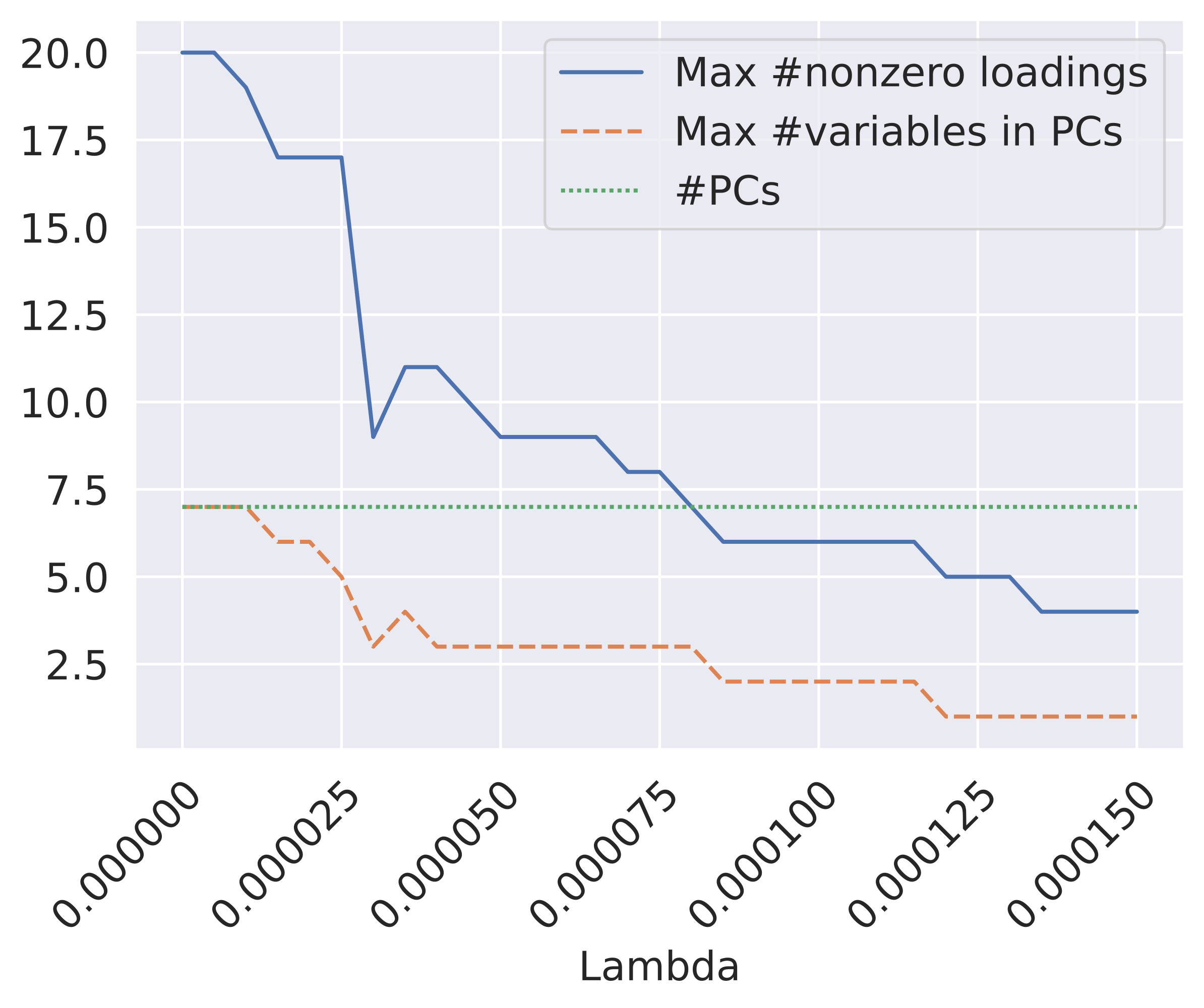
**

**Table S1. Adjusted hazard ratio per one standard deviation of each individual exposure using the Cox model**

| Individual exposures | Adjusted Hazard Ratio (95% CI) |
| --- | --- |
| Average Daytime Noise Level | 3.29 (0.00-inf^1^) |
| Average Evening Noise Level | 1.05 (0.00-1250691.42) |
| Average Night Time Noise Level | 0.29 (0.00-inf^1^) |
| Domestic Garden Percentage Within 1000M | 1.00 (0.95-1.06) |
| Domestic Garden Percentage Within 300M | 0.99 (0.94-1.05) |
| Greenspace Percentage Within 1000M | 1.01 (0.91-1.13) |
| Greenspace Percentage Within 300M | 0.99 (0.92, 1.07) |
| Natural Environment Percentage Within 1000M | 0.99 (0.91-1.08) |
| Natural Environment Percentage Within 300M | 1.00 (0.96-1.04) |
| Water Percentage Within 1000M | 1.00 (0.97-1.04) |
| Water Percentage Within 300M | 1.00 (0.96-1.03) |
| Coastal Distance | 1.00 (0.98-1.02) |
| NO_2_ | 1.00 (0.93-1.06) |
| NO_x_ | 1.01 (0.95-1.07) |
| PM_10_ | 1.00 (0.95-1.05) |
| PM_coarse_ | 1.00 (0.95-1.05) |
| PM_2.5_ | 1.00 (0.96-1.04) |
| Sum Of Major Roads Length Within 100M | 1.00 (0.97-1.03) |
| Traffic Intensity On Nearest Major Road | 1.00 (0.97-1.04) |
| Traffic Intensity On Nearest Road | 1.00 (0.97-1.03) |

^1^ represents extremely large values >1e10

*indicates statistical significance, i.e. 95%CI did not include 1.00.

**Figure S3. Pairwise Pearson correlation between socioeconomic, demographic, physiological and environmental factors in a large cohort of 379,690 in the UK**


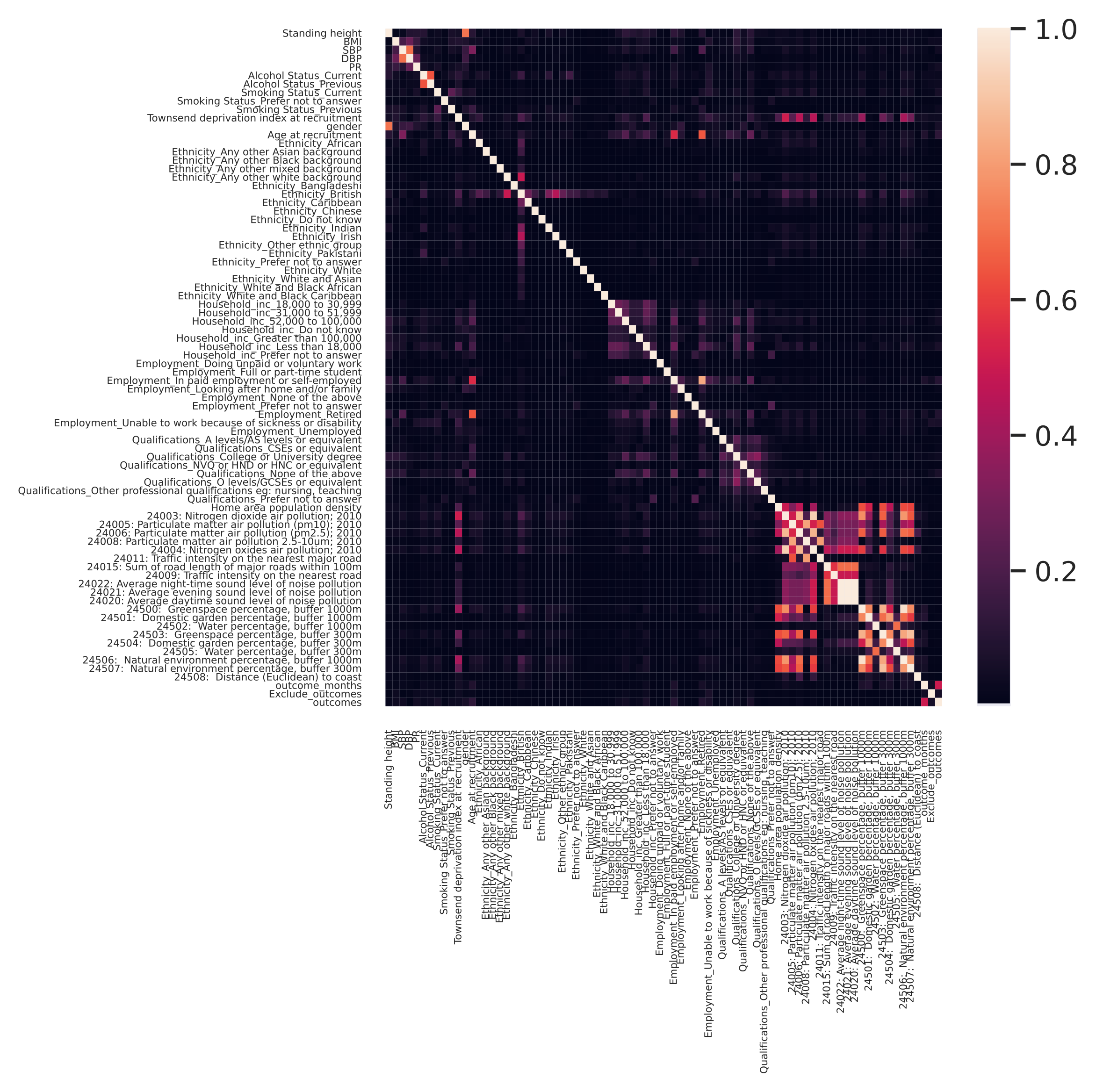


**Table S2. PCA Cox and SPCA Cox results**

| exposures | PCA Cox  Adjusted Hazard  Ratio (95% CI) | SPCA Cox  Adjusted Hazard  Ratio (95% CI) |
| --- | --- | --- |
| PC1 | 1.00 (1.00-1.01) | 0.99 (0.97-1.00) |
| PC2 | 1.01 (1.00-1.01) | 1.02 (1.00-1.03)^*^ |
| PC3 | 0.99 (0.98-1.00) | 1.01 (1.00-1.02) |
| PC4 | 1.01 (0.99-1.02) | 0.99 (0.96-1.01) |
| PC5 | 1.01 (0.99-1.03) | 0.98 (0.97-1.00) |
| PC6 | 0.99 (0.97-1.01) | 0.99 (0.98-1.01) |
| PC7 | 0.98 (0.95-1.00)^*^ | 1.01 (0.99-1.03 |

*indicates statistical significance, i.e. 95%CI did not include 1.00.

**Calculation of the hazard ratio in relation to average daily noise pollution**

Since,

PC2=0.577×(average daytime noise pollution+ average evening time noise pollution + average night-time noise pollution)

We rearrange this as

PC2=3 * 0.577 (average daily noise pollution) = 1.731 (average daily noise pollution)

The variables were normalised before the analysis. The standard deviation of the *average daily noise pollution* is 4.24 dB. Therefore, 4.240 dB/1.731=2.479 dB will increase the value of PC2 by 1 unit.

**Table S3. Exposure-response analysis for different noise pollution deciles compared to the lowest decile (46.23, 47.23]**

| daily sound pollution deciles [dB] | Cox  Adjusted Hazard  Ratio (95% CI) |
| --- | --- |
| **(47.23, 48.19]** | 1.05 (0.98-1.13) |
| **(48.19, 48.96]** | 1.04 (0.96-1.12) |
| **(48.96, 49.52]** | 1.07 (1.00-1.16)^*^ |
| **(49.52, 50.05]** | 1.08 (1.00-1.16) |
| **(50.05, 50.66]** | 1.05 (0.97-1.13) |
| **(50.66, 51.51]** | 1.10 (1.03-1.19)^*^ |
| **(51.51, 52.88]** | 1.09 (1.01-1.17)^*^ |
| **(52.88, 56.28]** | 1.08 (1.01-1.17)^*^ |
| **(56.28,88.53]** | 1.11 (1.01-1.22)^*^ |

*indicates statistical significance, i.e. 95%CI did not include 1.00.

**Table S4. Descriptive summary of the subpopulations in different noise pollution exposure categories**

| **daily noise pollution [dB]** | **(46.72,47.23]**  (*n* = 38,076) | **47.23,48.19]**  (*n* = 37,910) | **(48.19,48.96]**  (*n* = 38,178) | **(48.96,49.52]**  (*n* = 38,102) | **(49.52,50.05]**  (*n* = 37,589) | **(50.05,50.66]**  (*n* = 38038) |
| --- | --- | --- | --- | --- | --- | --- |
| Female (%) | 20892 (%54.87) | 20571 (%54.26) | 20830 (%54.56) | 20841 (%54.70) | 20479 (%54.48) | 20697 (%54.41) |
| Age, mean (SD) | 57.09 (7.96) | 57.01 (7.96) | 57.17 (7.97) | 57.18 (7.96) | 57.01 (7.97) | 56.83 (8.06) |
| Age at the time event, mean (SD) | 67.38 (7.15) | 67.21 (6.89) | 67.14 (6.99) | 67.28 (6.82) | 66.81 (7.08) | 66.79 (7.12) |
| Townsend deprivation index, mean (SD) | -2.05 (2.86) | -1.75 (2.99) | -1.89 (2.89) | -1.79 (2.82) | -1.52 (2.87) | -1.32 (2.93) |
| Ethnicity: British (%) | 90.6949 | 89.8417 | 89.7192 | 88.8772 | 88.055 | 88.1093 |
| Ethnicity: Any other white background (%) | 2.65784 | 2.88578 | 2.55907 | 2.93685 | 3.13656 | 3.12845 |
| Ethnicity: Irish (%) | 2.13258 | 2.25007 | 2.40453 | 2.60354 | 2.6843 | 2.63947 |
| Ethnicity: Indian (%) | 0.903456 | 1.03403 | 1.18131 | 1.38575 | 1.42595 | 1.27767 |
| Ethnicity: Carribean (%) | 0.606681 | 0.757056 | 0.851276 | 0.892342 | 1.01892 | 1.09101 |
| Ethnicity: other (%) | 1.66509 | 1.80427 | 1.94353 | 2.02614 | 2.14424 | 2.1978 |
| Annual average day-time noise level (dB(A))a, mean (SD) | 51.11 (0.13) | 51.89 (0.28) | 52.80 (0.22) | 53.44 (0.16) | 53.97 (0.15) | 54.54 (0.18) |
| Annual average evening noise level (dB(A))^a^, mean (SD) | 47.37 (0.13) | 48.15 (0.28) | 49.06 (0.22) | 49.70 (0.16) | 50.23 (0.15) | 50.79 (0.18) |
| Annul average night-time noise level (dB(A))^a^, mean (SD) | 42.29 (0.13) | 43.07 (0.28) | 43.98 (0.22) | 44.62 (0.16) | 45.15 (0.15) | 45.72 (0.18) |
| Domestic garden coverage (%) within 1000m^b^, mean (SD) | 23.86 (11.13) | 24.03 (10.78) | 25.91 (11.35) | 27.22 (11.17) | 27.07 (10.81) | 26.34 (10.65) |
| Domestic garden coverage (%) within 300m^b^, mean (SD) | 31.28 (14.55) | 31.35 (14.04) | 34.03 (14.48) | 35.95 (14.17) | 35.66 (13.67) | 34.40 (13.56) |
| Greenspace coverage (%) within 1000m^c^, mean (SD) | 49.88 (20.60) | 48.09 (20.10) | 46.12 (19.96) | 43.46 (19.70) | 42.24 (19.58) | 41.78 (20.13) |
| Greenspace coverage (%) within 300m^c^, mean (SD) | 39.66 (22.45) | 37.63 (21.61) | 35.38 (21.09) | 32.27 (20.51) | 31.01 (20.27) | 31.01 (20.88) |
| Natural environment coverage (%) within 1000m^d^, mean (SD) | 47.06 (24.51) | 44.54 (24.04) | 42.66 (23.71) | 39.77 (23.29) | 38.10 (23.24) | 37.24 (24.00) |
| Natural environment coverage (%) within 300m^d^, mean (SD) | 31.37 (25.11) | 28.36 (23.74) | 26.69 (22.72) | 24.13 (21.76) | 22.76 (21.53) | 22.46 (22.23) |
| Water body coverage (%) within 1000m^e^, mean (SD) | 1.24 (2.54) | 1.25 (2.48) | 1.17 (2.42) | 1.12 (2.35) | 1.08 (2.16) | 1.14 (2.34) |
| Water body coverage (%) within 300m^e^, mean (SD) | 0.96 (3.29) | 0.92 (3.07) | 0.83 (2.91) | 0.73 (2.56) | 0.67 (2.31) | 0.72 (2.48) |
| Costal distance (meter), mean (SD) | 46.16 (27.34) | 46.72 (26.81) | 47.09 (27.35) | 46.97 (26.94) | 46.35 (27.02) | 44.91 (27.07) |
| NO_2_; (𝜇g/m^3^), mean (SD) | 23.89 (6.02) | 24.80 (6.08) | 25.02 (5.85) | 25.74 (5.78) | 26.48 (5.95) | 26.98 (6.30) |
| NO_x_; (𝜇g/m^3^), mean (SD) | 37.17 (10.17) | 39.03 (10.35) | 39.56 (9.87) | 40.91 (9.65) | 42.59 (10.03) | 43.76 (10.76) |
| PM_10_; (𝜇g/m^3^), mean (SD) | 15.84 (1.81) | 15.89 (1.56) | 16.00 (1.62) | 16.08 (1.59) | 16.11 (1.53) | 16.12 (1.53) |
| PM_coarse_; (𝜇g/m^3^)^f^, mean (SD) | 6.30 (0.91) | 6.24 (0.74) | 6.28 (0.81) | 6.28 (0.81) | 6.27 (0.78) | 6.28 (0.76) |
| PM_2.5_; (𝜇g/m^3^), mean (SD) | 9.60 (0.84) | 9.77 (0.87) | 9.73 (0.82) | 9.81 (0.79) | 9.92 (0.81) | 10.01 (0.87) |
| Sum of major road length within 100m (m) ^g^, mean (SD) | 1.81 (17.52) | 14.68 (48.13) | 8.95 (41.74) | 5.10 (32.07) | 4.90 (31.09) | 5.29 (31.33) |
| Traffic intensity on nearest major road (vehicles/day)^h^, mean (SD) | 24054 (23040) | 21146 (18268) | 23247 (20271) | 23146 (19951) | 22483 (19142) | 22312 (18249) |
| Traffic intensity on nearest road (vehicles/day)^h^, mean (SD) | 669. (1710) | 1647(4408) | 1061 (3652) | 663 (2118) | 639 (2319) | 597 (1988) |
| Years of follow-up, mean (SD) | 8.09 (1.08) | 8.08 (1.08) | 8.02 (1.09) | 8.00 (1.11) | 8.01 (1.11) | 8.04 (1.10) |
| Number of events | 1380 (3.62%) | 1493 (3.94%) | 1436 (3.76%) | 1483 (3.89%) | 1492 (3.97%) | 1475 (3.88%) |
| Incidence rate, per 1000 person-years | 4 | 5 | 5 | 5 | 5 | 5 |

| **daily noise pollution [dB]** | **(50.66,51.51]**  (*n* = 37992) | **(51.51,52.88]**  (*n* = 37,947) | **(52.88,56.28]**  (*n* = 37,917) | **(56.28,88.53]**  (*n* = 37,941) |
| --- | --- | --- | --- | --- |
| Female (%) | 20744 (%54.60) | 20684 (%54.51) | 20731 (%54.67) | 20456 (%53.92) |
| Age, mean (SD) | 56.63 (8.06) | 56.62 (8.08) | 56.66 (8.04) | 56.65 (8.08) |
| Age at the time event, mean (SD) | 66.50 (7.27) | 66.80 (7.05) | 66.76 (6.98) | 66.69 (7.21) |
| Townsend deprivation index, mean (SD) | -1.20 (2.96) | -1.05 (3.01) | -0.92 (3.09) | -0.65 (3.30) |
| Ethnicity: British (%) | 87.7369 | 87.7724 | 87.5966 | 85.3193 |
| Ethnicity: Any other white background (%) | 3.227 | 3.39948 | 3.42063 | 3.84544 |
| Ethnicity: Irish (%) | 2.74268 | 2.60363 | 2.70064 | 2.78854 |
| Ethnicity: Indian (%) | 1.42925 | 1.28337 | 1.18417 | 1.69737 |
| Ethnicity: Carribean (%) | 1.07391 | 1.0989 | 1.15515 | 1.23613 |
| Ethnicity: other (%) | 2.26363 | 2.33484 | 2.2602 | 2.74637 |
| Annual average day-time noise level (dB(A))a, mean (SD) | 55.25 (0.24) | 56.33 (0.39) | 58.40 (0.95) | 65.95 (3.64) |
| Annual average evening noise level (dB(A))^a^, mean (SD) | 51.51 (0.24) | 52.58 (0.39) | 54.66 (0.95) | 62.21 (3.64) |
| Annul average night-time noise level (dB(A))^a^, mean (SD) | 46.43 (0.24) | 47.51 (0.39) | 49.58 (0.95) | 57.13 (3.64) |
| Domestic garden coverage (%) within 1000m^b^, mean (SD) | 24.77 (10.77) | 22.71 (11.03) | 20.63 (11.26) | 21.04 (11.25) |
| Domestic garden coverage (%) within 300m^b^, mean (SD) | 32.01 (13.92) | 28.59 (14.42) | 25.31 (14.60) | 25.29 (14.61) |
| Greenspace coverage (%) within 1000m^c^, mean (SD) | 42.78 (21.32) | 44.97 (23.23) | 47.77 (24.61) | 46.13 (23.55) |
| Greenspace coverage (%) within 300m^c^, mean (SD) | 32.62 (22.65) | 36.19 (25.39) | 40.04 (27.26) | 38.66 (25.65) |
| Natural environment coverage (%) within 1000m^d^, mean (SD) | 38.14 (25.52) | 40.52 (27.80) | 43.64 (29.46) | 41.64 (28.10) |
| Natural environment coverage (%) within 300m^d^, mean (SD) | 23.83 (24.60) | 27.50 (28.23) | 31.34 (30.65) | 28.77 (28.48) |
| Water body coverage (%) within 1000m^e^, mean (SD) | 1.22 (2.41) | 1.32 (2.59) | 1.43 (2.62) | 1.50 (2.57) |
| Water body coverage (%) within 300m^e^, mean (SD) | 0.80 (2.76) | 0.89 (2.88) | 1.06 (3.24) | 1.19 (3.18) |
| Costal distance (meter), mean (SD) | 44.71 (26.97) | 44.00 (26.49) | 44.01 (26.28) | 45.06 (25.39) |
| NO_2_; (𝜇g/m^3^), mean (SD) | 27.16 (6.78) | 27.11 (7.58) | 26.77 (8.15) | 32.98 (11.29) |
| NO_x_; (𝜇g/m^3^), mean (SD) | 44.49 (12.00) | 44.72 (13.88) | 44.48 (14.95) | 62.98 (26.83) |
| PM_10_; (𝜇g/m^3^), mean (SD) | 16.13 (1.66) | 16.13 (1.97) | 16.23 (2.28) | 17.73 (2.24) |
| PM_coarse_; (𝜇g/m^3^)^f^, mean (SD) | 6.34 (0.80) | 6.45 (0.91) | 6.61 (1.02) | 7.14 (0.92) |
| PM_2.5_; (𝜇g/m^3^), mean (SD) | 10.08 (0.98) | 10.11 (1.14) | 10.13 (1.25) | 10.67 (1.41) |
| Sum of major road length within 100m (m) ^g^, mean (SD) | 6.22 (35.16) | 9.08 (39.24) | 33.33 (69.15) | 187.79 (120.33) |
| Traffic intensity on nearest major road (vehicles/day)^h^, mean (SD) | 23169 (19487) | 24989 (22468) | 27782. (28195) | 22413 (21303) |
| Traffic intensity on nearest road (vehicles/day)^h^, mean (SD) | 634 (2794) | 670 (3262) | 837 (4366) | 7549(10831) |
| Years of follow-up, mean (SD) | 8.05 (1.11) | 8.05 (1.13) | 8.07 (1.10) | 8.06 (1.11) |
| Number of events | 1548 (4.07%) | 1538 (4.05%) | 1543 (4.07%) | 1608 (4.24%) |
| Incidence rate, per 1000 person-years | 5 | 5 | 5 | 5 |
